# Multidimensional Interventions on Supporting Disease Management for Hospitalized Patients with Heart Failure: The Role of Clinical and Community Pharmacists

**DOI:** 10.3390/jcm12083037

**Published:** 2023-04-21

**Authors:** Magdalena Jasińska-Stroschein, Magdalena Waszyk-Nowaczyk

**Affiliations:** 1 Department of Biopharmacy, Medical University of Łódź, ul. Muszyńskiego 1, 90-151 Lodz, Poland; 2 Pharmacy Practice Division, Chair and Department of Pharmaceutical Technology, Poznan University of Medical Sciences, 6 Grunwaldzka Street, 60-780 Poznan, Poland

**Keywords:** heart failure, pharmacist-led intervention, re-admission, adherence, disease management, meta-research

## Abstract

Background: existing trials on the role of clinical pharmacists in managing chronic disease patients have focused on variety of interventions, including preparing patients for the transition from hospital to home. However, little quantitative evidence is available regarding the effect of multidimensional interventions on supporting disease management for hospitalized patients with heart failure (HF). The present paper reviews the effects of inpatient, discharge and/or after-discharge interventions performed on hospitalized HF patients by multidisciplinary teams, including pharmacists. Methods: articles were identified through search engines in three electronic databases following the PRISMA Protocol. Randomized controlled trials (RCTs) or non-randomized intervention studies conducted in the period 1992–2022 were included. In all studies, baseline characteristics of patients as well as study end-points were described in relation to a control group i.e., usual care and a group of subjects that received care from a clinical and/or community pharmacist, as well as other health professionals (Intervention). Study outcomes included all-cause hospital 30-day re-admission or emergency room (ER) visits, all-cause hospitalization within >30 days after discharge, specific-cause hospitalization rates, medication adherence and mortality. The secondary outcomes included adverse events and quality of life. Quality assessment was carried out using RoB 2 Risk of Bias Tool. Publication bias across studies was determined using the funnel plot and Egger’s regression test. Results: a total of 34 protocols were included in the review, while the data from 33 trials were included in further quantitative analyses. The heterogeneity between studies was high. Pharmacist-led interventions, usually performed within interprofessional care teams, reduced the rates of 30-day all-cause hospital re-admission (odds ratio, OR = 0.78; 95% CI 0.62–0.98; *p* = 0.03) and all-cause hospitalization >30 days after discharge (OR = 0.73; 95% CI 0.63–0.86; *p* = 0.0001). Subjects hospitalized primarily due to heart failure demonstrated reduced risk of hospital admission within longer periods, i.e., from 60 to 365 days after discharge (OR = 0.64; 95% CI 0.51–0.81; *p* = 0.0002). The rate of all-cause hospitalization was reduced by multidimensional interventions taken by pharmacists: reviews of medicine lists and/or their reconciliation at discharge (OR = 0.63; 95% CI 0.43–0.91; *p* = 0.014), as well as interventions that were based mainly on patient education and counseling (OR = 0.65; 95% CI 0.49–0.88; *p* = 0.0047). In conclusion, given that HF patients often have complex treatment regimens and multiple comorbid conditions, our findings highlight the need for greater involvement from skilled clinical and community pharmacists in disease management.

## 1. Introduction

The prognosis for patients with heart failure (HF) has improved considerably in the last few decades; however, it remains unsatisfactory. Five-year mortality rates are high, ranging from 20 to 67%; quality of life (QoL) is also markedly reduced [1]. The prevalence of HF appears to be up to 2% of adults, representing about 5 per 1000 person-years [1]. After initial diagnosis, HF patients are hospitalized once every year on average. Due to population growth and ageing, the absolute number of hospital admissions for HF is expected to increase considerably in the future (potentially by as much as 50% in the next 25 years) [1]. This phenomenon is also supported by the fact that HF patients, especially those with HF with preserved ejection fraction (HFpEF), exhibit a large number of comorbidities, including hypertension (HTN), renal dysfunction, diabetes mellitus and/or obesity affecting the entire cardiovascular system [2]. Acute coronary syndromes (ACS), rapid arrhythmias and severe bradycardia/conduction disturbance can also be specific causes of heart failure, hospitalization and urgent emergency visits [2,3]. About 20% of patients with HF are affected by chronic obstructive pulmonary disease (COPD), which has a major impact on symptoms and outcomes [4]. Medication and dietary non-adherence have also been recognized as contributing factors in up to 30% of hospitalized HF patients [5].

Clinical and community pharmacists are recognized as important actors in the heath care system. They contribute to the management of chronic diseases, including hypertension, diabetes mellitus, hyperlipidemia or anticoagulation therapy [6]. Many studies have focused on the role of pharmacists in the setting of heart failure. Such approaches should be multifactorial due to the incidences of comorbidities in HF, e.g., ischemic heart failure or hypertension, polypharmacy, potential drug–drug interactions and the presence of patient-related specific determinants of pharmacotherapy, such as age, renal or hepatic function, etc. [7,8]. Clinical pharmacists are capable of performing in-patient medication reviews (MRs) and reconciliations at discharge and, thus, assisting the physician in optimization of pharmacotherapy. Other tasks could include drug therapy monitoring and education about the disease, therapy and lifestyle modifications. In some diseases, patient education and drug counseling is crucial for medication adherence. This can in turn correspond with a patient’s clinical condition, thereby determining the hospital readmission [9].

A disease management program led by a HF specialist nurse, as a part of multidisciplinary team, is strongly recommended in the updated clinical guidelines [2]. The question about the role of clinical pharmacists in such multidisciplinary teams remains open. A number of prospective and retrospective studies have been conducted with the purpose of evaluating the benefits of pharmacist-led interventions on in-patient and after discharge management of heart failure. The results of individual clinical trials are diverse and ambiguous. Although recent systematic reviews have evaluated the role of the pharmacist in multidisciplinary approaches for patients hospitalized for heart failure and resulting potential improvements in their outcomes after discharge [10,11,12,13], the results of pooled quantitative analysis aiming to compare the benefits of such activities are scarce.

The study presents a systematic review and quantitative analysis of data from randomized and non-randomized controlled trials by evaluating the effects of various interventions taken by pharmacists for patients suffering from HF. Pharmacists could work in co-operation with other health care providers. Different end-points were also analyzed. The primary outcomes included those associated with rates of hospitalization and death, as well as medication adherence. The secondary outcomes were quality of life, prescription of relevant medications, medication errors and adverse events. Where possible, a cut-off point of 30 days was used when assessing the risk of hospital re-admission. The contribution of such additional factors (variables) was also taken into account; variables included he follow-up period and type of pharmacist-led intervention, as well as key clinical characteristics, such as diagnosis on admission, comorbidities, age, New York Heart Association functional class (NYHA-FC) and left ventricular ejection fraction (LVEF%).

## 2. Materials and Methods

The systematic review was conducted in accordance with the Cochrane guidelines, including the statement on Preferred Reporting Items for Systematic Review and Meta-analysis (PRISMA) (Supplementary Table S1).

### 2.1. Search Strategy

Three databases (PubMed/Medline: 1992–2022; Ebsco: 1992–2022 and Embase: 1992–2022) were searched and the following criteria were considered: (“Patient Discharge” OR “discharge” OR “hospitalization” OR “hospitalized”), (“pharmacist” OR “pharmacy” OR “pharmaceutical care”) and (“heart failure” OR “cardiac failure”).

### 2.2. Selection of Studies

For the purposes of study selection, the following PICO criteria were defined: randomized controlled trials or non-randomized intervention studies performed on adult patients from any country who were hospitalized with diagnosis of HF (P—population). The intervention (I) must have targeted the management of heart failure, e.g., medication review, education and counseling in cooperation with other health professionals; a health service intervention might have also aimed to prepare patients for the transition from hospital to home (reconciliation at discharge, education, monitoring, or supporting the patient in the post-discharge phase in hospital or at home). The comparator (C) was the Usual Care Group (control group), while outcomes (O) (see Section 2.5) were reported for a minimum follow-up of 30 days. Baseline characteristics for patients and study end-points were described in relation to a control group, as well as a group of subjects that received care from a clinical and/or community pharmacist. Pharmacists could work together with other health professionals. Trials that did not fulfil the defined criteria were not included. The study selection was completed independently by two reviewers (M.J.-S., M.W.-N.). Observational retrospective studies, review papers and conference posters were not included.

### 2.3. Extraction of Data and Assessment of Study Quality

The extracted data included population characteristics (age, sex, admission diagnosis, comorbidities, criteria for HF, NYHA classification, values of LVEF%, if possible), descriptions of the intervention, study end-points and items needed for the assessments of study quality. Standardized forms for extracted data were used. The quality of an individual study was estimated in accordance with the revised version of Cochrane Collaboration’s RoB 2 Risk of Bias Tool [14,15]. Two reviewers (M.J.-S., M.W.-N.) assessed the risk of bias independently and any differences were solved by discussion to reach consensus.

### 2.4. Analysis of Study Data

As the overall effect size, the odds ratio (OR) with 95% confidence interval (CI) was adjusted for dichotomous data. The calculations concerned the individual study outcomes reported as a proportion of hospitalized or deceased patients according to intention-to-treat analysis. The percentage of adherent patients, as defined by each study, was also considered. Due to expected high heterogeneity between studies, a random-effects model was proposed. Heterogeneity was estimated using the χ^2^ test (I^2^). Another measure used was Cochran’s Q statistics. The heterogeneity between two sub-groups of patients was designated by statistically pronounced Q values (*p* < 0.05). In order to evaluate the influence of each study on the overall effect size, sensitivity analysis was conducted using the leave-one-out method, i.e., removing one study each time and repeating the analysis. Bias across the studies was estimated using Duval and Tweedie’s trim and fill method with Egger’s test. The analyses were conducted using STATISTICA 13.1 software and R Core Team (RoB 2 Risk of Bias Tool) [15]. A *p*-value < 0.05 was considered statistically evident.

### 2.5. Study Outcomes

Study end-points (outcomes) included all-cause hospital 30-day re-admission or emergency room (ER) visits, hospitalization within a period longer than 30 days after dis-charge, as defined by each study (e.g., 60-, 90-, 180- or 365-day period), specific-cause hospitalization rates, mortality (primary), time to first unplanned hospitalization, adverse events, medication adherence, quality of life and health literacy (secondary end-points).

## 3. Results

### 3.1. Search Results and Characteristics of Individual Studies

**Figure 1:**
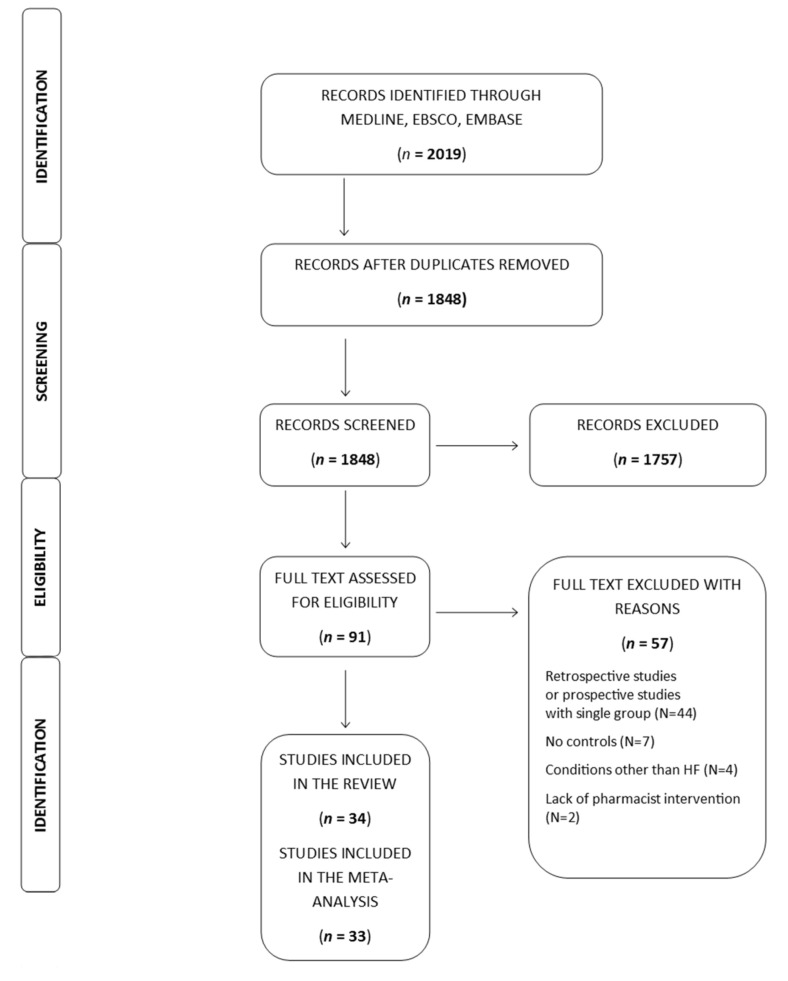
PRISMA flow chart of study search.

The search included 2019 titles. During the process of study selection, 91 papers were indicated to be relevant to the review question and full-text articles were assessed for the purposes of the present review. Finally, the systematic review included 34 papers, as presented in the PRISMA flow chart in Figure 1. One study assessed the potential advantages of pharmacist-driven intervention, albeit only in relation to underutilization of cardiovascular medications [16]. Ultimately, 33 studies were included as part of a further meta-analysis. The number of participants was 12,048, while the mean ages was 66.7 years (62.8–70.6) (Intervention) and 64.1 years (61.2–67.0) (Usual care). The proportion of women was 48%.

### 3.2. Quality Assessments

#### 3.2.1. Randomization—Bias

50% of trials demonstrated unclear risk of bias (some concerns). These protocols did not report the methods used to generate random sequence. Moreover, the process of allocation of participants was not described. The randomized allocation of subjects to the study groups was not performed in 29.4% protocols (high risk of bias).

#### 3.2.2. Deviations from the Intended Interventions—Bias

In some protocols (open trials), the subjects knew about the intervention (29.4%). In some trials, no information was provided about blinding (44.2%). In 44.2% of studies, it was not clear whether data were analyzed according to the intention-to-treat principle. In total, 5.9% of trials did not provide a consort flow diagram; thereby, it was not possible to follow the participants in a study. Overall, the risk of bias in 64.7% of studies was judged as unclear, while 32.4% of studies were highly biased.

#### 3.2.3. Missing Data (Outcomes)—Bias

26.5% of protocols demonstrated unclear risk of bias (some concerns). Some trials presented high proportion of missing data (ranging from 10 to 30%), while the proportion of missing data varied between study groups (32.3%).

#### 3.2.4. Measurement of Outcomes—Bias

In most cases, the information about blinding was not provided and/or it was not known whether the research evaluations were determined by the participants’ knowledge of the study groups to which they belonged (61.8%). In 38.2% of the included studies, it was stated that the outcome assessors knew about the intervention or that the trials were open. This phenomenon could have determined a final outcome e.g., where patients were asked to report their quality of life or adherence to treatment. In 71% of papers, the risk of bias was assessed as unclear (some concerns).

#### 3.2.5. Selection of the Result—Bias

In 23.5% of papers, the study result being evaluated was likely to have been adjusted from multiple data analyses. This was true for the measurement of parameters such as quality of life or adherence (different scales, dichotomous or continuous data). A total of 76.5% of protocols demonstrated low risk of bias.

**Figure 2:**
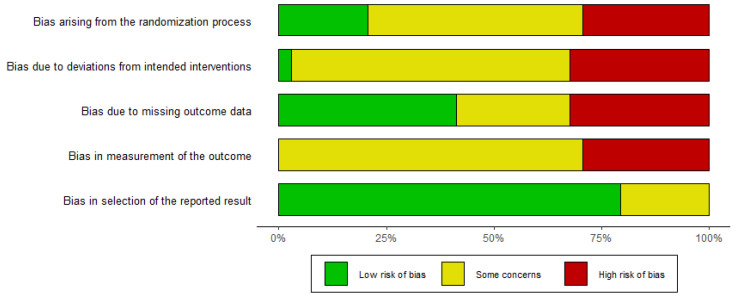
Results of risk of bias assessments according to RoB 2 Risk of Bias Tool [15].

In the case of *n* = 2 (5.9%) studies, the risk of bias was assessed as low, while *n* = 17 (50%) protocols were judged as unclear (some concerns); *n* = 15 (44.2%) studies were highly biased. Figure 2 summarizes the results of bias assessments.

The results of sensitivity analysis are presented in Supplementary Figure S1. Statistical significance was not influenced by any single study included in the meta-analysis according to the all-cause hospitalization rate. In relation to the all-cause mortality parameter, the analyses were repeated after removing one study [17].

Further analyses were performed to assess publication bias. The analyses concerned outcomes such as all-cause mortality and all-cause hospitalization rates. The sub-group analyses were performed by baseline diagnosis in the intervention arm, type of pharmacists intervention and follow-up. In most cases, the absence of publication bias was designated by a symmetrical plot and a non-significant (NS) result of Egger’s test, as demonstrated in Table 1.

### 3.3. Study Characteristics

The review included 34 prospective intervention studies (including 26 randomized controlled trials) (Table 2). In 18 papers, all subjects were hospitalized for heart failure, as defined by each protocol. The inclusion criteria were mainly subjects with HFrEF where LVEF was below 40 or 45%; in some papers, the baseline diagnosis was defined as congestive heart failure. Patients with HF and preserved ejection fraction (HFpEF) were also included. Only 30% of papers included in the review provided data regarding the patients’ classification according to NYHA-FC. In the remaining trials (*n* = 16 studies), heart failure was listed as one of the comorbidities. This group also experienced related conditions, such as arterial hypertension (HTN), cerebrovascular diseases (CVD) with stroke, arrhythmias, acute coronary syndrome (ACS), coronary artery disease (CAD), diabetes mellitus (DM), dyslipidemia or chronic obstructive pulmonary disease (COPD). Table 2 summarizes the pharmacist-led interventions focused on HF patients; subjects assigned to the usual care group received the services typically provided by an individual center, as defined for each protocol. In 28/34 studies, clinical pharmacists took care of HF patients in co-operation with other health care providers (e.g., general practitioners, medical specialists, nurses, and/or community pharmacists). In 19 studies (55.8%), clinical pharmacists’ activities focused on in-patient medicine management and consultation to identify drug-related problems (DRPs), reviews of medicine lists, and reconciliation at discharge. Pharmacists assessed patient knowledge about the disease, treatment, possible side-effects and lifestyle changes that could help control symptoms and added information where necessary; they also provided patient motivation after discharge. In 14/34 studies, pharmacists mainly prepared patients for the transition from hospital to home through counselling and education. In six/34 studies, the patient-oriented activities could also have been performed by community pharmacists. They received notes about the hospitalization and any medication-related issues identified or needed upon discharge; post-discharge medication reviews were performed in order to optimize medical treatment. They could also conduct home-based visits. In one study, pharmacist transitions coordinators worked with in-patient and community-based pharmacists.

Table 3 summarizes the educational interventions that were performed by pharmacists in the reviewed protocols.

In *n* = 22 studies (64.7%), the beneficial effects of pharmacists led-interventions for hospitalized patients were assessed for at least a 30-day follow-up period; a more pronounced parameter was the rate of re-hospitalizations. Some authors (*n* = 14 studies; 41.2%) did not consider 30-day follow-ups in their observations and reported outcomes for longer periods, i.e., 60, 90, 180 or 365 days after discharge. In general, the median follow-up period was 90 days (IQR 30–180 days) after discharge.

### 3.4. Synthesis and Analysis of Study Data

The most frequently reported data in individual trials was included in the meta-analysis’ these data included all-cause hospital 30-day re-admission rates, all-cause >30-day hospitalization rates, all-cause mortality and medication adherence. The effects of pharmacist-led intervention on the time taken up by the first unplanned hospitalization, health literacy, quality life, adverse events or clinical outcomes for individual studies are given in Table 2.

In general, pharmacist-led interventions reduced the rate of 30-day all-cause hospital re-admission (OR = 0.78; 95% CI 0.62–0.98; *p* = 0.03) (*n* = 16 studies) and the rate of all-cause hospitalization more than 30 days after discharge (OR = 0.73; 95% CI 0.63–0.86; *p* = 0.0001) (*n* = 19 studies).

The rate for hospitalizations of patients admitted with diagnoses such as heart failure, ACS, HTN, dyslipidemias or diabetes was insignificantly decreased in the Intervention Group as compared to the Usual Care Group (OR = 0.76; 95% CI 0.57–1.02; *p* = 0.06) for 30-day and (OR = 0.85; 95% CI 0.70–1.03; *p* = 0.1) >30-day follow-up periods.

**Figure 3:**
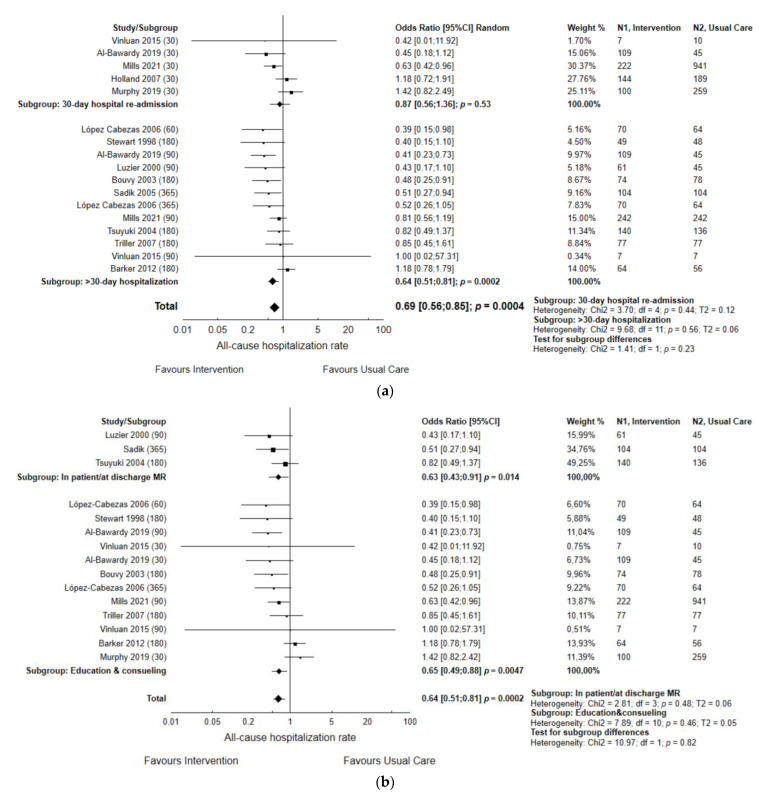
Forest plot comparing intervention vs. usual care for dichotomous data concerning hospitalization rate (odds ratio, OR) in studies where 100% of patients were hospitalized from heart failure. (**a**)—all cause hospitalization, including 30-day re-admission, according to the number of days before the next admission to hospital. Pharmacist-led interventions caused a significant reduction in the risk of hospitalization within >30 days after discharge. (**b**)—all cause hospitalization according to type of pharmacist-led intervention. The first sub-group (in-patient/at discharge MR) received services that were focused on in-patient medication reviews and/or reconciliation at discharge, while the second group was prepared for the transition from hospital to home, mainly by counselling and education. Both types of intervention significantly reduced the risk of hospital admission. Days of follow-up are shown in brackets [3,18,19,22,26,33,34,37,40,43,44,45,47].

Further analyses based on the random-effects model identified an insignificant reduction in 30-day re-admission rates, primarily among patients primarily hospitalized for heart failure. In this sub-group of patients, the risk of >30-day hospitalization was significantly decreased (*p* = 0.0002) (Figure 3a).

Figure 3b demonstrates the influence of the type of intervention led by pharmacists. The activities that focused on in-patient medication reviews and/or reconciliation at discharge and included mainly education and counseling tended to decrease rates of hospitalization (*p* < 0.05).

In 11 studies, the efficacy of interventions taken by multidisciplinary teams was assessed in relation to all-cause hospitalization rates (30-day, or >30 days after discharge). The additional analysis aimed to compare the effects of care with clinical pharmacists as a part of such a multidisciplinary team. The risk of hospitalization was more significantly reduced (OR = 0.80; 0.65–0.99; *p* = 0.037; T^2^ = 0.025; I^2^ = 49.11%) in the Intervention Group compared to the Usual Care Group (*n* = 11 studies). The included patients were hospitalized due to heart failure and other conditions. For a more homogenous sub-group of patients, i.e., 100% of subjects hospitalized for HF, the result was non-significant (*n* = 5 studies) (OR = 0.86; 0.65–1.14; *p* = 0.28; T^2^ = 0.06; I^2^ = 50.26%). Due to study heterogeneity (follow-up, primarily diagnosis) this finding needs to be verified with a higher number of protocols.

Due to the limited number of studies accompanied with pronounced heterogeneity, it was not possible to perform further sub-group analyses addressing specific-cause hospitalization rates e.g., associated with cardiac-related or DRP issues.

**Figure 4:**
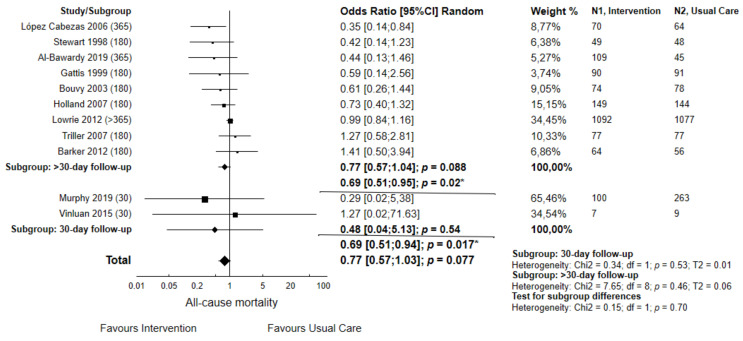
Forest plot of comparison: intervention vs. usual care for dichotomous data concerning mortality (odds ratio, OR) in studies where 100% of patients were hospitalized from heart failure-related conditions. Pharmacist-led interventions provided a slight reduction in mortality (*p* = 0.077). Days of follow-up are shown in brackets. * Outcomes after removing one study according to the results of sensitivity analysis [3,18,19,20,23,25,26,33,43,44,47].

Figure 4 demonstrates the rate of death in hospitalized patients with heart failure receiving pharmacist-led interventions compared to usual care. These activities only demonstrated slight beneficial impacts on patients and mortality. Non-RCT studies demonstrated lower risk of mortality in patients diagnosed from HF compared to RCT protocols (OR = 0.42; 95% CI 0.19–0.91; *p* = 0.03 vs. OR = 0.91; 95% CI 0.74–1.10; *p* = 0.32) (heterogeneity Chi2, *p* = 0.06).

The risk of death among patients admitted with heart failure, ACS, HTN, dyslipidemias or diabetes was non-significantly decreased in the Intervention Group compared to the Usual Care Group (OR = 0.92; 95% CI 0.56–1.51 *p* = 0.74). Due to the limited number of studies, the comparative analysis concerning follow-up period, i.e., 30-day vs. >30-day mortality, could not be performed.

**Figure 5:**
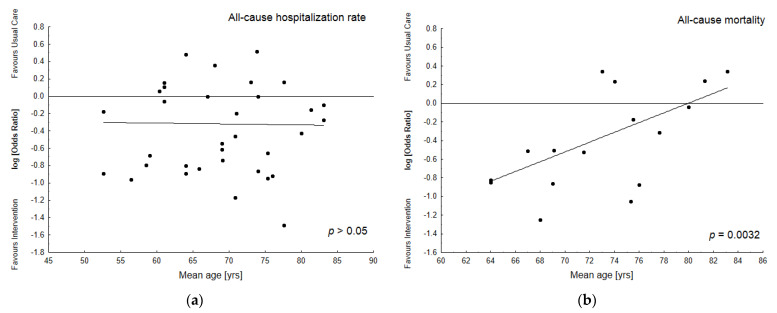
Bubble plots with fitted meta-regression lines of effect size demonstrate alterations in odds ratio (OR) for the particular outcome as a function of the mean age of subjects in the Intervention Group. (**a**)—all cause hospitalization (NS); (**b**)—all cause mortality (*p* = 0.0032) [3,18,19,22,24,25,26,33,34,37,39,40,41,43,44,45,47].

Figure 5 demonstrates the insignificant influence of another covariate (age) for all-cause hospitalization rates.

A meta-analysis for medication adherence was performed for dichotomous measures (*n* = 5 studies). The findings indicate the percentage of patients taking more than 80% of the prescribed doses (>80% compliance) [19,39] or prescription filling [46]. In one study, a score of BMQ-Regimen Screen ≥1 indicated potential non-adherence [20]. In five studies, all patients were hospitalized for HF. In general, medication adherence was not improved in studies where interventions were performed within multidisciplinary teams (OR = 2.62; 95% CI 0.68; 10.01; *p* > 0.05) rather than usual care.

The authors hope to analyze the impact of pharmacist-led interventions in potentially reducing adverse drug events (adverse drug reactions), as well as improving the health literacy or quality of life. Unfortunately, it was not possible to perform the pre-defined calculations due to the limited number of studies and pronounced heterogeneity (follow-up, admission diagnosis).

**Figure 6:**
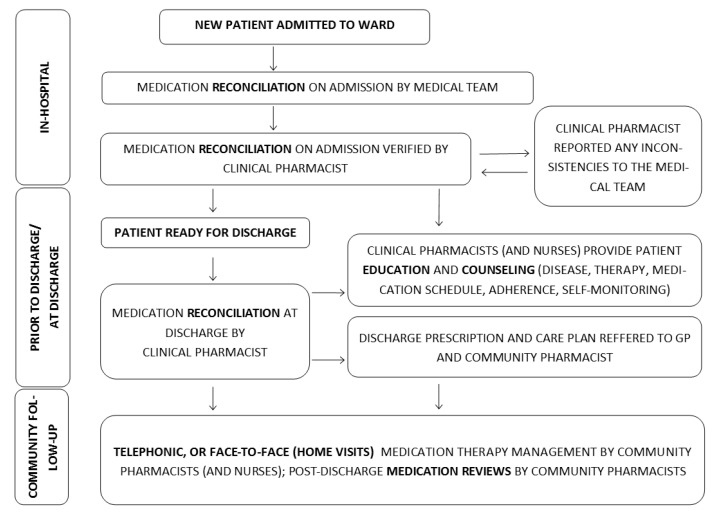
A flow chart describing process of comprehensive care and role of clinical and community pharmacist.

Figure 6 summarizes the role of pharmacists in an interprofessional care team, according to trial protocols reviewed in the present paper.

## 4. Discussion

The present study reviews prospective studies on the effectiveness of pharmacist-led interventions for heart failure; it is recognized as baseline diagnosis at admission against a range of outcomes and performs a pooled analysis. The most frequent outcome assessed in the studies was the risk of all-cause readmission. This risk was based on a cut-off point of 30 days, where possible; this cut-off point was selected due to the hospital readmissions reduction programs implemented in some countries, including the USA, which penalize hospitals with excess in-patient rehospitalizations within 30 days of index in-patient stays for targeted conditions [49].

### 4.1. Hospitalization Rate and Mortality

The pooled analysis of data from 26 studies, which compared patients receiving a number of pharmacist-led interventions to those receiving usual care, revealed reductions in the rates of all-cause 30-day hospital re-admission and >30-day hospitalization. The sub-group of patients primarily hospitalized for heart failure alone received some benefits, which manifested as a reduced risk of hospitalization more than 30 days after discharge. However, this was not the case among the more heterogeneous group of patients, i.e., those hospitalized for HF and other conditions. These patients displayed a non-significant reduction in hospitalization rate for the 30-day and longer follow-up period (i.e., from 60 to 365 days). A very recent systematic review qualitatively assessed the rates for hospital readmissions, especially at the 30-day follow-up stage [12]. Patients were admitted with primary diagnosis of acute myocardial infarction, pneumonia, heart failure, COPD, diabetes, dysrhythmias and other internal diseases. The review, which includes 37 RCTs and non-RCTs, found that pharmacist-led interventions that include communication with a primary care physician were effective in reducing readmissions. However, the performed analyses did not concern the HF-patient sub-group.

A meta-analysis by Van Spall et al. (2017) compared the effectiveness of transitional care services in decreasing all-cause death and all-cause readmission rates following hospitalization for heart failure based on 53 RCT studies [13]. In contrast to the current study, some interventions were performed by various health care professionals. The authors classified services into the following categories: education alone, telemonitoring, telephone support (without remote telemonitoring), nurse home visits (clinical assessment and education) and a combination of nurse home visits with structured telephone support and follow-up visits at a hospital. A final category—pharmacist interventions—concerned visits by pharmacists for education, medication reconciliation and optimization after discharge according to data reported in four trials. Among the services that significantly decreased all-cause re-hospitalization and mortality compared with usual care, nurse home visits were most effective. Telephone, telemonitoring, pharmacist, and education interventions did not significantly improve clinical outcomes.

Despite the diverse and multi-component structure of interventions performed by pharmacists in the trials included in the current analysis, it was possible to distinguish two sub-groups of patients. The first sub-group received services that were focused on in-patient medication reviews and/or reconciliation at discharge, while the second group was mainly prepared for the transition from hospital to home through counselling and education. Some authors indicated that the latter program can be more readily adapted to a variety of “real life” settings suitable for patients with HF [45]. The present study demonstrates the efficacy of such activities; rates for hospitalization were reduced significantly. In general, clinical pharmacists reconciled pre-admission and at-discharge medications with the patient and reported any inconsistencies to the medical team prior to hospital discharge. They also could contact physicians regarding drug therapy if rationalization of therapy or simplification of dosage regimens was appropriate. Educational interventions varied in individual protocols and could be performed prior to and after discharge. They were based on the tailored HF management programs, supported by written or audiovisual materials and adherence aids for patients. The emphasis was placed on simple language, adapted to the social and cultural level of the patient [33]. In a majority of studies, the benefits of patient education were reported for longer follow-ups of typically three-to-six months, which was probably due to a whole range of positive factors that came about as a result of the multi-component interventions. It could be hypothesized that intervention patients tended to seek help more frequently at the casualty department, perhaps indicating a better understanding of the need for early medical intervention in management their heart failure when symptoms were deteriorating. This view was supported by phenomena where the nature of intervention additionally involved home-based pharmacist visits or contacts with community pharmacists who were familiarized with the discharge medication list.

The mortality rates were assessed in *n* = 17 studies. For most sub-groups of patients and follow-up periods, the interventions performed by pharmacists did not reduce the rates of death from any cause. The obtained results are also similar to those reported in reviews including populations of patients hospitalized for a wide spectrum of chronic disorders [50].

### 4.2. Medication Adherence

Undoubtedly, medication adherence among HF patients is a continual challenge. Few strategies for reducing DRPs, including medication non-adherence, in patients hospitalized for heart failure have been presented in the existing literature. In a very recent systematic review, Hernández-Prats et al. (2022) summarized the available evidence resulting from interventions led by pharmacists aimed at reducing inappropriate medication prescription in patients with heart failure [11]. The authors conclude that evaluating the suitability of treatment to specific HF guidelines can be crucial. Other papers concerned chronically ill subjects without addressing their particular disorders. For example, Kelly et al. (2021) assessed the relationship between pharmacist medication counseling, medication adherence, 30-day hospital readmission rates and mortality [50]. The authors included 62 RCTs; in most studies, the participants were older patients with various chronic diseases and polypharmacy. Pharmacist medication counseling was associated with a statistically significant 30% increase in relative risk for medication adherence and a 24–30% risk reduction in 30-day hospital readmission rates or emergency department visits.

In the present study, due to different scales and follow-ups the pooled analysis of dichotomous data was performed for only five studies, where all patients were hospitalized for heart failure. In general, medication adherence was not improved by interventions being performed within multidisciplinary teams compared to usual care; however, these findings need to be verified by a higher number of studies.

### 4.3. Quality of Life and Health Literacy

Better medication adherence should be accompanied by increased health literacy; such improved knowledge was reported among HF patients in some trials. This could concern items such as knowledge of weight control, self-adjustment of diuretics, signs and symptoms of heart failure and side-effects of drugs [31,46]. This disorder and its symptoms can greatly affect the ability of patients to perform normal daily activities. Some studies focused on the potential role of the pharmacist in improving quality of life among patients hospitalized for heart failure. In general, the results indicated that benefits were obtained in both the Intervention and Usual Care sub-groups. Various measures of quality of life were used, including SF-36 scores, Minnesota Living with Heart Failure (MLHF), health visual analogue scale (VAS) and EUROQOL, EQ-5D and Assessment of Quality of Life instrument (AQoL). Only in a study by Varma et al. (1999) did patients receiving pharmacist-led interventions tend to score lower (improved QoL) on the MLHF questionnaire throughout the study; the only significant difference was at 9 months (*p* = 0.04) [46]. However, as quality of life (QoL) was reported by means of a variety of scales and follow-ups, more detailed comparative analyses could not be performed. In isolated studies, QoL or health literacy assessments were accompanied by an assessment of patient satisfaction score [33,37].

### 4.4. Practical Implications

In trials that were included in the systematic review, interprofessional teams consisted of clinical pharmacists and other health care providers. As discussed previously, clinical pharmacists can successfully contribute to such teams, offering effective and safe care [51]. This can include optimization of medical therapy or dealing with drug interactions or medicine side-effects, as employed by the health care systems in the USA, the UK, Canada or New Zealand [52]. Indeed, most of the clinical studies were conducted in the USA (20/34) and the UK (4/34). Moreover, in other European countries, such as Norway and Sweden, 5/34 trials showed that the need for clinical pharmacy services and agreements to perform these services in hospitals has increased substantially in recent years. Nevertheless, the structure of the multicomponent service and the degree of cooperation between clinical pharmacists and other health care professionals may represent important limitations in the present paper. Another point is the extent of transmission of patient information from the in-patient setting to the community pharmacy. This point might be key to reducing fragmentation in health care and empowering patients with knowledge about their disease state and therapy; this approach provides them with a sense of self-efficacy, which further encourages positive lifestyle changes and medication adherence [53].

### 4.5. Strengths and Limitations

Although the present study was not restricted to RCTs, the comparative analyses did not reveal any significant differences between RCT and non-RCT studies according to rate of hospitalization. Little publication bias was observed across the studies. As mentioned earlier, most of the reviewed studies were conducted in the USA and UK. This phenomenon seems to indicate the substantial role of clinical pharmacy services in the USA and UK. Sub-group analyses were performed where possible and several variables/factors that could impact the final outcome were identified. This included the participation of patients with heart failure in primary admission diagnosis. If possible, the results were (re)calculated according to intention-to-treat analysis. The main outcomes reported in the individual studies—no matter if they were included into meta-analysis or not—are summarized in Table 2.

Despite its strengths, the review has some limitations. The first limitation is the high risk of bias, as shown in the case of 44.2% of trials. The trials also demonstrated high heterogeneity with regard to the intervention type and its multicomponent structure (e.g., in-patient medication review and/or conciliation, at discharge, education and counselling, post-discharge visits at home, contacts with community pharmacists, etc.), follow-up periods and diverse measures for outcomes. For example, due to differences in the scales and measures (dichotomous or continuous data, lower/higher score-related increases in an individual parameter, etc.), it was not possible to perform analyses in relation to QoL. In addition, due to incomplete reporting, it was possible to perform neither comparative analyses regarding the inter alia relationship between medication adherence and hospitalization rate in HF patients, nor assess the impact of clinical characteristics on final outcomes. Indeed, regressive analyses were only performed for mortality and age. For the same reason, the specific-cause hospitalization rates, i.e., cardiac- or DRP-related rates, were not assessed. Another point is that patient-oriented activities were performed by specific HF pharmacists in none of 34 studies. In most trials, clinical pharmacists took care of participants with specialists in geriatrics, cardiology, internal medicine and/or specialist heart failure nurses. Further trials are needed to evaluate the advantages of HF pharmacist-driven interventions in reducing the hospitalizations related to heart failure. As mentioned above, a key “interpretative” limitation of the present study can be the degree of cooperation between clinical pharmacists and other health care professionals, as well as the extent of transmission of patient information from the in-patient setting to the community pharmacy in “real life”.

## 5. Conclusions

In conclusion, given that patients with heart failure often have complex treatment regimens and multiple comorbid conditions, while HF accounts for 2–5% of hospital admissions, the obtained results point to the need for greater involvement of skilled clinical and community pharmacists in disease management. Only the multicomponent interventions that includes both in-patient medication reviews and reconciliation at discharge and post-discharge follow-up with the contribution of community pharmacies, as well as patient education and counseling at each of these stages, could produce some benefit. These benefits could be associated with decreased hospitalization rates, including 30-day re-admission rates.

## Figures and Tables

**Table 1:** Results from publication bias funnel plot and trim and fill analysis.

Parameter—Odds Ratio [95% CI]	Admission Diagnosis in the Intervention Arm	Pharmacists Intervention	Follow-Up	Egger’s Regression *p*-Value	Imputed (Trim and Fill) *
All-cause mortality	All patients	All types	All	NS	6
			30-day	NS	0
			>30-day	NS	4
	Sub-group: HF, only		All	0.028	1
	Sub-group: HF and other conditions			NS	2
All-cause hospitalization	All patients	All types	All	0.001	2
			30-day	0.015	6
			>30-day	NS	0
	Sub-group: HF, only		All	NS	0
	Sub-group: HF and other conditions			NS	4
	All patients	Sub-group: In-patient/at discharge MR	All	0.0036	1
		Sub-group: Education and counseling		0.019	2

HF—heart failure; NS—non significant. * trim and fill method included the following procedures: (1) trim (removing) the smaller studies causing funnel plot asymmetry; (2) using the trimmed funnel plot to estimate the true ‘center’ of the funnel; and (3) replacing the omitted studies and their missing ‘counterparts’ around the center (filling). The first column from the right provides an estimate of the number of missing (omitted) studies. Non-significant Egger’s test results indicate the absence of publication bias across the studies for the particular set of variables, e.g., tested parameter; all-cause mortality; admission diagnosis in the intervention arm for all patients; pharmacist intervention: all types; and follow-up more than 30 days after discharge.

**Table 2:** Study characteristics.

First Author’s Name/Year/ Location	Study Type, Sample Size #, Follow Up (Days)	Admission Diagnosis * (%)	Comorbidities	Intervention Performed by Pharmacist (Key Components)	End-Points (Intervention vs. Control Group) ^
Al-Bawardy, R. 2019 USA [18]	NRS 109/45 (365)	HF (100%)	-	Early post-discharge HF medication review (MR) as “Brown Bag”.	All-cause hospitalization (*p* = 0.09); mortality (NS)
Barker, A. 2012 Australia [19]	RCT 64/56 (180)	HF (100%)	AF, DM, HTN, COPD	Education about incorporating medication into daily routines; verification of patient follow-up appointment with doctor; identification of expired medications and their disposal; contacts with the community pharmacist to ensure continuity of the medication regimen.	Number and days of all-cause (*p* = 0.002) and HF-related hospitalizations (NS), mortality (NS); AQoL (NS); SF-36 (NS)
Bell, S.P. 2016 USA [20]	RCT 423/428 (30)	HF (31%)	ACS, CAD, CBD, DM, Dys, HTN	Reconciliation of preadmission and discharge medications, pre-discharge reporting about any inconsistencies to the medical team. Medication schedules showing the discharge regimen, and a pillbox, education, were provided. Dealing with barriers to medication adherence, and troubleshooting barriers while the patient was in the hospital and at discharge	Time to first unplanned health care event (NS); all-cause hospitalization or ER visit (NS)
Bloodworth, L.S. 2019 USA [21]	RCT 96/160 (180)	HF (30%)	ACS, COPD, PN	Pre-discharge medication reconciliation and 30 days of medications on discharge provided by a pharmacist transitions coordinator (PTC) who worked with inpatient and community-based pharmacists. After discharge community pharmacists provided telephonic and face-to-face medication therapy management	All-cause hospital re-admission (30-day; *p* = 0.033); time to first unplanned re-admission (NS)
Bouvy, M.L. 2003 The Netherlands [22]	RCT 74/78 (180)	HF (100%)	Arrhythmia, CKD, HTN, DM, COPD	Patient education about drug use, reasons for noncompliance, possible adverse drug reactions and difficulties to integrate medication use in daily life, providing reports to the GP. After discharge, community pharmacist contacted patients on a monthly basis for a maximum of 6 months	All-cause hospitalization (100% HF; *p* = 0.024); medication adherence (*p* = 0.05); mortality (NS); QoL (*p* = 0.02)
Eggink, R.N. 2010 Sweden [23]	RCT 41/44 (30)	HF (100%)	-	Identification of potential prescription errors in the discharge medication in order to discuss them with the cardiologist. Verbal and written information about (side)effects of, and changes in hospital drug therapy upon hospital discharge were provided. A discharge medication list (including dose adjustments, discontinued medication) was approved by the physician, faxed to the community pharmacy and given as written information to the patient with the instruction to hand it over to their GP.	Number of medication discrepancies after discharge (*p* = 0.01); medication adherence (NS)
Farris, K.B. 2014 USA [24]	RCT 314/316 (90)	HF (27.3%)	Asthma, CAD, depression, DM, Dys, fracture, HTN, stroke	Medication reconciliation during admission, education during in-patient stay, discharged counseling (goals of therapy, medication administration, barriers to adherence including cost and patient concerns) and discharged medication list. A telephone call was received at 3–5 days post-discharge, while a discharge care plan focused on medication changes and recommendations was given to the GP and community pharmacist.	All-cause hospitalization (NS); AEs (NS)
Gattis, W.A. 1999 USA [25]	RCT 90/91 (180)	HF (100%)	-	Discharge medication reviews, therapeutic recommendations to the physician, patient education, and follow-up telemonitoring.	All-cause mortality (NS); composite of all-cause mortality and non-fatal HF (*p* = 0.005)
Gustafsson, M. 2017 Sweden [26]	RCT 212/217 (180)	HF (34%)	Arrhythmia, COPD, DM, HTN, malignant disease, stroke	Medication reconciliation, including medication list, list of laboratory results, medical record notes from primary care and index admission, etc. Identification of relevant DRPs with respect to impairment of body function (renal function, liver function, contraindications, allergies, swallowing problems), certain drug use (toxic drugs, drugs prone to produce side effect, potentially inappropriate drugs), interactions (drug–drug, and drug–food), symptoms (adverse drug reactions) and general judgment of the patient’s drug use (proper drug selection, dosage, duration of treatment, polypharmacy, indication for therapy, untreated indication, adherence, over-the-counter drugs and effectiveness).	All-cause (NS), DRP-related (*p* = 0.03) hospitalization; mortality (NS); ↓ PIMs (*p* = 0.01)
Heaton, P.C. 2019 USA [27]	RCT 213/187 (30)	HF (28.6%)	ACS, COPD	Medication reconciliation, comprehensive medication review, patient counseling and disease-state education on new medication or diagnoses. Self-management education and set health-related goals for patients using motivational interviewing techniques were provided. Identification of medication non-adherence.	All-cause hospital re-admission (*p* = 0.054)
Holland, R. 2007 UK [28]	RCT 149/144 (180)	HF (100%)	-	Two home visits within two and eight weeks of discharge were provided by community pharmacists. MRs and lifestyle advice (basic exercise, dietary, and smoking cessation advice) was also provided. Completion of simple sign and symptom monitoring diary cards (including monitoring body weight) was encouraged, while recommendations were fed back to the general practitioner and local pharmacist.	All-cause hospitalization (NS); mortality (NS); EQ-5D (*p* = 0.08)
Israel, E.N. 2013 USA [16]	RCT 241/246 (90)	HF (27.1%)	ACS, CAD, Dys, HTN	Comprehensive medication reconciliations in order to identify drug-related problems within 24 h of admission were provided. Recommendations to the in-patient care team and to patients’ community physicians were; a discharge care plan containing the patient’s discharge medication list and recommendations to optimize regimens were also provided.	Cardiovascular medication underutilization (30, 90 days after discharge; NS)
Kebede, A.T. 2022 Norway [29]	RCT 19/20 (90)	HF (25%)	AF, CAD, CHF, COPD, HTN, osteoporosis, chronic respiratory failure	Inhaler technique training during the hospital stay and upon discharge was offered by the postgraduate pharmacy students. In case of observed critical errors, the attending hospital physicians were informed. The postgraduate students could suggest a change in COPD treatment, such as adding or discontinuing an inhaler medication or changing the inhaler device, when deemed appropriate based on treatment guidelines. The decision to implement the suggested changes was made by a physician.	All-cause hospitalization (NS); CAT-score (NS)
Kripalani, S. 2012 USA [30]	RCT 423/428 (30)	ACS or HF (100%)	CAD, CVD, DM, Dys, HTN, MI, PCI, stroke	Pharmacist-assisted medication reconciliation, in-patient pharmacist counseling, low-literacy adherence aids, and individualized telephone follow-up support after discharge were provided.	Medication errors after hospital discharge (*p* = 0.0001); AEs (NS)
Linné, A.B. 1999 Sweden [31]	RCT 64/66 (180)	HF (100%)	CAD, DM, Dys, HTN, PCI	Patient counselling and education about basic facts on diuretics, digitalis, ACE-inhibitors, nitrates and low-dose aspirin were provided. Leaflets containing general information on effects and side effects of these drugs were given on an individual basis.	HF literacy; difference in knowledge–tested by questionnaire after 6 months (*p* = 0.005)
Lisenby, K.M. 2015 USA [32]	NRS with historical control 43/65 (30)	HF (28%)	COPD, DM	Medication reconciliation, therapeutic recommendations, patient discharge counseling and a post-discharge follow-up phone call was provided. Each medication list was scanned for drug interactions, pertinent allergies and duplications; the results were appropriately addressed by the medical team.	All-cause hospital re-admission (NS); time from discharge to readmission (NS)
López Cabezas, C. 2006 Spain [33]	RCT 70/64 (365)	HF (100%)	AF, CABG, DM, HTN, PCI, RD	Patient discharge counseling and education on the disease, diet and drug therapy were provided.	Mean days of hospital stay per patient (at 60 and 180 days; *p* < 0.05); all-cause hospitalization (at 60 days after discharge *p* < 0.05); mortality (*p* = 0.02); EUROQoL (NS); satisfaction score (at 60 days; *p* = 0.026)
Lowrie, R. 2012 UK [17]	RCT 1092/1077 (4.7 years)	HF (100%)	AF, CABG, DM, HTN, MI, PCI, RD, stroke	Post-discharge medication reviews were provided in order to optimize medical treatment for left ventricular systolic dysfunction by community pharmacists.	Composite of death (NS) or hospitalization for worsening HF after discharge (NS)
Luzier, AB. 2000 USA [34]	NRS 51/31 (90)	HF (100%)	CAD, CKD, DM, HTN, COPD	In-patient and discharge medication reviews were provided, while attending physicians offered counseling with recommendations for medical optimization changes in drug doses to optimize therapy.	All-cause hospitalization (after 90 days *p* = 0.07)
Makowsky, M.J. 2009 Canada [35]	qRCT 220/231 (180)	HF or COPD (100%)	CAD, DM, PN	Medication history, patient-care round participation, resolution of drug-related issues and discharge counseling were provided.	All-cause hospitalization (90-day; *p* = 0.025); QoL (*p* < 0.05)
McKinley, D. 2019 USA [36]	NRS with historical control 74/58 (30)	HF (47.3%)	Anemia, arrhythmia, DM, Dys, heart attack, HTN, obesity, renal disease	Medication reconciliation was provided in order to optimize a patient’s medication regimen within the multidisciplinary team; consultations on the appropriateness of prescribed medications also occurred.	All-cause hospital re-admission (*p* = 0.045); ↑ in beta-blocker prescriptions and ↓ in medications that should be avoided in HF.
Mills, A.A. 2021 USA [37]	NRS with historical control 222/941 (90)	HF (100%)	Anemia, arrhythmia, CAD, CKD, DM, Dys	Education on disease state, diet and medication education was provided by a pharmacy student.	All-cause hospital re-admission (*p* = 0.04); patient-reported satisfaction score (NS)
Murphy, J.A. 2019 USA [3]	NRS with historical control 100/259 (30)	HF (100%)	COPD, DM, HTN	In-patient and discharge pharmacist counseling and education, as well as a post-discharge follow-up phone call, was provided. Upon discharge, the pharmacist helped ensure the patient had a follow-up appointment with their cardiologist within one week.	All-cause hospital re-admission (NS); mortality (NS)
Njonkou, G. 2021 USA [38]	NRS 167/60 (30)	HF (2.4%)	AF, anticoagulation, deep vein thrombosis, DM, Dys, HTN, COPD	Medication dose titrations, pharmacotherapy optimization, medication reconciliation, disease state counseling and screening for medication-related problems was provided following evidence-based protocols.	% of patients with therapeutic goal (INR—NS, HbA1c—*p* = 0.024, BP—NS); all-cause hospital re-admission (*p* = 0.0064)
Roblek, T. 2016 Slovenia [39]	RCT 26/25 (180)	HF (64%)	CKD, DM, Dys, HTN, stroke	In-patient and discharge advice relating to the clinically relevant drug–drug interactions was provided. Recommendations were sent to GPs (discharge letters).	Incidence of drug–drug, all-cause hospitalization (NS); mortality (NS)
Sadik, A. 2005 UK [40]	RCT 104/104 (365)	HF (100%)	CAD, Cardiomyopathy, DM, HTN, Mitral valve disease	Pharmacists contacted physicians regarding drug therapy if rationalization of therapy or simplification of dosage regimens was deemed appropriate. Patient education (in the form of a printed booklet contained information on HF, its symptoms, the aims of treatment, the types of medication used and their possible side-effects, diet and lifestyle changes, advice to stick to one brand of digoxin and information on the action to take if doses of medication were missed) was provided.	All-cause hospitalization (*p* = 0.03); medication adherence (*p* < 0.05); MLHFQ (*p* < 0.05); SF-36 (NS)
Schnipper, J.L. 2010 USA [NCT00632021] [41]	RCT 423/428 (30)	ACS or acute HF (100%)	CAD, DM, Dys, HTN, PCI, stroke	Medication reconciliation, initial counseling, discharge counseling and follow-up phone calls (1 to 4 days after discharge) were provided.	Unplanned hospitalization and ER visit (NS); serious medical events (NS); mortality (NS)
Sjölander, M. 2019 Sweden [42]	RCT 212/217 (180)	HF (34%)	AF, DM, HTN, malignant dis, stroke, COPD	Medication review and reconciliation and participation in ward rounds. The patients’ drug lists were updated and then reviewed by the pharmacists with special attention paid to DRPs. Clinically relevant DRPs were discussed at ward rounds.	Drug-related hospitalization (30-day; *p* = 0.038)
Stewart, S. 1998 Australia [43]	RTC 49/48 (180)	HF (100%)	AF, CAD, COPD, HTN, DM, MI	After discharge, a single home visit was provided by a nurse and pharmacist to optimize medication management, identify early clinical deterioration and intensify medical follow-up and caregiver vigilance as appropriate.	All-cause hospitalization (*p* = 0.075); mortality (NS)
Triller, D.M. 2007 USA [44]	RCT 77/77 (180)	HF (100%)	-	Initial comprehensive in-home medication assessment and two follow-up visits were provided. Throughout the three-week period, the clinical pharmacist accessed and reviewed all pertinent physician notes and laboratory test values and interacted with prescribers on behalf of the patients as necessary.	All-cause (NS) and HF-related (NS) hospitalization; mortality (NS);
Tsuyuki, R.T. 2004 Canada [45]	RCT 140/136 (180)	HF (100%)	AF, CAD, CVD, DM, Dys, HTN, MI, PVD	Verbal counseling was provided to the study patients; advice on medication reviews, education about disease, medication adherence, lifestyle, non-drug treatment and self-monitoring was also provided. Follow-up consisted of telephone contact by the local research coordinator monthly for 6 months after discharge and aimed to reinforce education and adherence relating to the disease.	All-cause (NS) and cardiac-related hospitalization (*p* = 0.02); total length of hospital stay (days) (*p* < 0.005); medication adherence (NS)
Varma, S. 1999 UK [46]	RCT 42/41 (365)	HF (100%)	COPD, DM, PN	Education on the disease, treatment, and lifestyle changes that could help control symptoms was provided. Patients also were encouraged to monitor their symptoms and comply with prescribed drug therapy.	Medication knowledge score (*p* < 0.05); QoL (*p* = 0.04)
Vinluan, C.M. 2015 USA [47]	RCT 7/9 (90)	HF (100%)	Arrhythmia, CKD, DM, HTN	Individualized in-patient counselling by a pharmacist and telephone call follow-up with a review of current medications and HF counselling after discharge was provided at day 3, 30, 60 and 90 (pathogenesis, HF symptoms, information on medication therapy and lifestyle education).	All-cause hospitalization (NS); medication adherence (NS); mortality (NS)
Wright, E.A. 2019 USA [48]	Pragmatic intervention 615/3075 (30)	HF (71%)	COPD, DM, PN	Initial medication reconciliation was provided; in-patient pharmacists completed a summary of the encounter with the use of a standardized template, which included the discharge medication list, allergy information, vaccination history, principal hospital diagnosis, active problem list, recent laboratory results, post-discharge appointments, primary care and nurse management, etc. Pharmacists provided notes directly to the community pharmacist about the hospitalization and any medication-related issues identified or requiring intervention upon discharge.	All-cause hospital re-admission (NS); mortality (*p* = 0.037)

#—intervention/usual care; *—Intervention Group; ^—statistical significance of the result for particular interventions led by pharmacist denoting decrease in risk of hospitalization (mortality, adverse reactions) or improvements in QoL or medication adherence; ACS—acute coronary syndrome; AE—adverse event; AF—atrial fibrillation; BP—blood pressure; CABG—coronary artery bypass graft; CAD—coronary artery disease; CAT—COPD Assessment Test; CKD—chronic kidney disease; COPD—chronic obstructive pulmonary disease; CVD—cerebrovascular disease; DM—diabetes mellitus; Dys—dyslipidemia; ER—emergency room; HF—heart failure; HTN—hypertension; MLHFQ—Minnesota Living with Heart Failure Questionnaire; NRS—non-randomized controlled study; NS—non-significant; PCI—percutaneous coronary intervention; PIM—potentially inappropriate medications; PN—pneumonia; PVD—peripheral vascular disease; RCT—randomized controlled trial; and RD—respiratory disease. The contribution of community pharmacists is underlined.

**Table 3:** Summary of educational interventions according to reviewed papers.

Stage	Intervention/Tool	Details	References
Prior to discharge and at discharge	Patient education	(1) the disease and symptoms (e.g., early recognition of worsening symptoms, knowing when to call the physician, how to respond to escalating signs); (2) self-monitoring (e.g., salt and fluid restriction, daily weighing); (3) exercise alternating with rest periods; (4) rationale for medical therapy, proven benefits, indications and contraindications and adverse reactions; (5) secondary lifestyle modifications (e.g., blood pressure and glycaemia control, alcohol and smoking cessation, etc.)	[16,18,20,30,34,37,40,42,45,46]
	Medication schedule	(1) education on proper medication use; an illustrated schedule showing the discharge regimen (e.g., proper use of diuretics in relation to peripheral edema); (2) information on action to take if doses of medication are missed; (3) identification and avoidance of medication errors	[4,19,20]
	Adherence aids	(1) pillbox with the patient practiced filling; (2) personalized illustrated medication schedule	[27,37,41]
	Materials	(1) booklets and leaflets (e.g., written, audiovisual or available for downloading from the websites); (2) interactive photo CD program	[18,20,31,33,34,37,40,45,46]
	Event diary	(1) symptom monitoring diary cards (including monitoring body weight)	[34,40]
After discharge	Systematic phone calls	(1) reinforcement of education; (2) management with barriers to medication adherence	[30,45,46]

## Data Availability

Authors can confirm that all relevant data are available in the article’s Supplementary Materials and on request from the corresponding authors.

## Supplementary Materials

The following supporting information can be downloaded at: https://www.mdpi.com/article/10.3390/jcm12083037/s1, Figure S1: Leave-one-out sensitivity analysis. (a)—all-cause hospitalization rate; (b,c)—all-cause mortality (before and after excluding of one study) [3,18,19,22,26,33,34,37,40,43,44,45,47]; Table S1: PRISMA 2020 Checklist.
